# Antrodia camphorata polysaccharide resists 6‐OHDA‐induced dopaminergic neuronal damage by inhibiting ROS‐NLRP3 activation

**DOI:** 10.1002/brb3.1824

**Published:** 2020-09-09

**Authors:** Chenyang Han, Heping Shen, Yi Yang, Yongjia Sheng, Jin Wang, Wenyan Li, Xiaohong Zhou, Li Guo, Liping Zhai, Qiaobing Guan

**Affiliations:** ^1^ Department of Pharmacy The Second Affiliated Hospital of Jiaxing University Jiaxing China; ^2^ Department of Neurology The Second Affiliated Hospital of Jiaxing University Jiaxing China

**Keywords:** 6‐OHDA, Antrodia camphorata polysaccharide, dopaminergic neuronal, Parkinsons disease

## Abstract

**Introduction:**

Parkinson's disease (PD) is a common degenerative disease of the central nervous system (CNS). The main pathological change is the apoptosis of dopaminergic neurons in the substantia nigra pars compacta (SNPc), thereby leading to dopamine reduction in nigral striatum. 6‐Hydroxydopamine (6‐OHDA), a neurotoxic substance, mediates apoptosis of dopaminergic neurons and causes Parkinson‐like symptoms in mice.

**Methods:**

Our team previously found that Antrodia camphorata polysaccharide (ACP) exerted a good behavioral improvement effect on the PD mouse model established by 6‐OHDA; however, the mechanism remains unknown. Therefore, in this study, we focused on ROS‐NLRP3 signal to investigate the mechanism of 6‐OHDA‐induced apoptosis of dopaminergic neurons MES23.5 and the protective effects of ACP on dopaminergic neurons.

**Result:**

6‐OHDA could further activate the expression of inflammasome NLRP3 by inducing ROS, thereby resulting in apoptosis of MES23.5 cells. ACP could inhibit the expression of ROS‐NLRP3 induced by 6‐OHDA, exerting a protective role in MES23.5 cells. Animal experiments also confirmed that ACP intervention could reduce the activation level of ROS‐NLRP3 in the substantia nigra–striatum and improve the exercise capacity of PD mice.

**Conclusion:**

Our study validated that 6‐OHDA could induce apoptosis of dopaminergic neurons via ROS‐NLRP3 activation. ACP could inhibit this signal and protect dopaminergic neurons, which might be promising in research of PD therapeutics.

## Graphical abstract

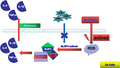

## 1 INTRODUCTION

Neurodegenerative disease is defined as degenerative diseases of the nervous system caused by degeneration and necrosis of neurons, mainly including Alzheimer's disease (AD) and Parkinson's disease (PD). PD is the second most common neurodegenerative disease, with its incidence second only to AD (Kochergin & Zakharova, 2016; Saraiva et al., 2016). PD most frequently occurs in the elderly population. The main pathological changes of PD include abnormal protein aggregation in the dopaminergic neurons in the substantia nigra of the midbrain, leading to degeneration and necrosis of neurons, deposition of eosinophilic inclusion body (Lewy body), thereby causing reduced dopaminergic neurons, and progressive reduction of dopamine transmitters (Mercado, Castillo, Soto, & Sidhu, 2016; Yamasaki et al., 2016). The pathogenesis of PD is complicated. A large number of studies have found that neuroinflammation plays an important role in PD. The main characteristics of PD neuroinflammation are the activation of microglia in the midbrain SNc region and the release of a large number of inflammatory factors (Hirsch & Hunot, 2009; Hirsch, Vyas, & Hunot, 2012). The release of various inflammatory factors, such as IL‐1β and IL‐18, is associated with the activation of inflammasome. NOD‐like receptors (NLRs) are the most studied PRR recognition receptors. After activation of NLRs, a large protein complex‐inflammasome is formed. Among them, NLRP3 inflammasome is the most widely studied (Mao et al., 2017; Sznejder‐Pachołek, Joniec‐Maciejak, Wawer, Ciesielska, & Mirowska‐Guzel, 2016). Godolo et al (van de Weg et al., 2015) have found that the activation of NLRP3 is involved in a variety of neurodegenerative diseases. Lu et al (Zhu, Li, & Lu, 2014) also report that MPP^+^ stimulation of astrocytes can further activate NLRP3, supporting its role in PD. However, the current study has not further explained the upstream mechanism of NLRP3 activation. Mitochondrial dysfunction is the main marker of oxidative stress injury, which can lead to excessive production of ROS. ROS mainly act on oxidative stress damage and activate various downstream signals. ROS has been found to be involved in neuroinflammation and nerve injury in PD research. PINK1 and Parkin genes can stabilize mitochondrial function and inhibit ROS damage, while ROS can activate NLRP3 (Letteria et al., 2016). It remains unclear whether there is cascade reaction in ROS‐NLRP3 in the progression of PD.

Antrodia camphorata polysaccharide (ACP) is the main component of the natural polyporaceae Aphididae (Liu et al., 2004). ACP is composed of a variety of monosaccharides and is a mixture. The concentration of ACP in the experiment is above 95%. In our previous study, we have found that that ACP exerts a good regulatory role on neuroinflammation, which can regulate the activation of NLRP3, and the release of downstream inflammatory factors, with a good behavioral regulatory effect on the PD mouse model. In consideration of the wide regulatory effect of ROS on neuroinflammation, herein, in this study, we mainly focused on dopaminergic neurons and ROS‐NLRP3, and investigated the role of ROS‐NLRP3 in dopaminergic neurons and the mechanism of ACP in protecting neurons.

## 2 MATERIALS AND METHODS

### 2.1 Experimental design and cell grouping

Dopaminergic neuron cell line MES23.5 (Shanghai Sur Biotech Co., Ltd.) was used in this study. (a) In studying 6‐OHDA‐activated ROS‐induced NLRP3 activation, MES23.5 was divided into control group (Con group) and 6‐OHDA group. To be specific, MES23.5 in Con group was conventionally cultured, while MES23.5 in 6‐OHDA group was intervened with 6‐OHDA at a final concentration of 50 mmol/L (IC50). (b) ROS inhibitor NAC was used to pretreat MES23.5 cells to inhibit ROS production. Cells then were divided into 6‐OHDA and 6‐OHDA + NAC groups (NAC is a ROS inhibitor), both of which were treated with 150 µM 6‐OHDA (IC50). Cells in OHDA + NAC group were pretreated with NAC at a concentration of 10 mmo/L. (c) In order to explore whether ACP could intervene ROS production, MES23.5 was categorized into control group (Con group), 6‐OHDA group, 10 mmo/L ACP group, 20 mmo/L ACP group, and 50 mmo/L ACP group. Cells in Con group were conventionally cultured, while cells in the other four groups were treated with 50‐mm/L 6‐OHDA. In addition, cells in ACP intervention groups were pretreated with 10, 20, and 50 mmo/L ACP.

### 2.2 Cell viability by CCK‐8 assay and the determination of 6‐OHDA dose

MES23.5 cells were cultured in DMEM medium supplemented with 5% FBS in poly‐L‐lysine‐coated flasks. When cells grew into logarithmic phase, cells were digested and inoculated into 96‐well plates (100 µl/per well). After cell attachment, drug was added for intervention. The pretreatment time of both NAC and ACP was 3 hrs. Three hours after pretreatment, the medium was discarded, and the medium containing 6‐OHDA was added for further incubation for 24 hr. After the intervention, 10 µl of CCK‐8 solution (Beyotime Biotechnology Co., Ltd.) was added into each well for incubation for another 4 hrs after mixture. Blank medium was set as the control. After the incubation, the absorbance value OD was determined at 450 nm. Cell viability was calculated after subtracting the blank control. In the IC50 value test of 6‐OHDA, MES23.5 cells were treated with 6‐OHDA at a final concentration of 5, 10, 20, 40, 80, and 160 mmol/L for 24 hr. Afterward, cell viability of each group was calculated, followed by plotting standard curves to calculate the IC50 value.

### 2.3 Detection of intracellular ROS level using DCFH‐DA probe

DCFH‐DA probe (Beyotime Biotechnology Co., Ltd.) was used to detect intercellular ROS. In brief, cells were seeded into 6‐well plates and intervened with drug for 24 hr. Afterward, medium was discarded, DCFH‐DA probe was diluted with serum‐free medium at a ratio of 1:1,000, followed by addition of 1 ml medium containing the DCFH‐DA probe into each well. After incubation for 30 min, the medium was discarded, and cells were washed twice with serum‐free medium. The cell staining was observed under a fluorescence microscope, and the absorbance was measured by a fluorescence spectrophotometer.

### 2.4 Determination of intercellular ROS level by flow cytometry

Cells were seeded into 6‐well plates and incubated with drugs for 24 hr. Then, cells were collected to adjust cell concentration to 10^6^/ml, followed by staining with the above‐described DCFH‐DA probe for 30 min. Cells were washed twice with serum‐free medium after the staining, followed by detection of ROS expression by flow cytometry.

### 2.5 The expression level of cellular protein by Western blot assay

Cells were collected after drug intervention for 24 hr, washed twice with PBS, and then lysed in 1.0 ml of RIPA lysis buffer (Beyotime Biotechnology Co., Ltd.) for 30 min on ice. After centrifuging at 10,000 *g* for 15 min, supernatant was collected, followed by protein concentration detection by BCA kit (Beyotime Biotechnology Co., Ltd.). According to the molecular weight, 8%–12% SDS‐PAGE gel was separately collocated. 5× loading buffer was used to supplement the protein sample to 20 µl. Protein samples were boiled for 8 min and underwent electrophoresis at 80 V, which subsequently converted to 120 V and transferred to PVDF membrane under 300 mA constant for 0.5‐2 hr. The PVDF membrane was blocked in 5% nonfatty milk powder for 2 hrs. Primary antibodies against NLRP3, ASC, caspase‐1, and pro‐caspase‐1 were diluted into 1:500, 1:500, 1:600, and 1:450 (Abcam) in TBST. After incubation with appropriate primary antibodies, the PVDF membrane was washed twice with TBST and incubated with horseradish peroxidase‐labeled goat anti‐rabbit secondary antibody (Abcam) (dilution: 1:20,000). After the incubation, the chemiluminescence method was used to detect the optical density using Image Pro‐Plus 6.0 software. GAPDH was used as the internal control. Results were shown as the optical density values of comparison between the target protein and the internal control protein.

### 2.6 The expression levels of IL‐1β and IL‐18 in the cell culture medium measured by enzyme‐linked immunosorbent assay (ELISA)

ELISA kit (Abcam) was used, and the standard curve was plotted according to the manufacturer's instruction. After 24 hr of drug intervention, cells were collected, washed twice with PBS, and added with 1.0 ml of RIPA lysis (Beyotime Biotechnology Co., Ltd.) on ice for 30 min. After centrifuging at 10,000 *g* for 15 min, supernatant was collected to determine protein concentration using BCA kit (Beyotime Biotechnology Co., Ltd.). After adjusting the protein concentration, protein levels were measured using a microplate reader according to the instructions. The standard curve was used to calculate the expression level of cytokines.

### 2.7 Cell apoptosis by flow cytometry

Cells were harvested 24 hr after drug intervention, and adherent cells were digested with trypsin without EDTA. After washing twice with PBS, cell concentration was adjusted to 5 × 10^5^/ml, followed by addition of 500 µl of binding buffer, 5 µl of Annexin‐FITC, and 5 µl of propidium iodide. After incubation for 15 min in dark, cell apoptosis was measured by flow cytometry.

### 2.8 The expression of key mRNA using real‐time quantitative PCR (RT‐qPCR)

Total RNA was extracted using TRIzol after cells were treated with drug for 24 hr. In brief, appropriate amount of TRIzol reagent was added and homogenized for 2 min, followed by centrifugation at 12,000 r/min for 5 min to discard the precipitate. Chloroform was added in an amount of 200 µl/per ml of supernatant. After shaking for 15 s, the mixture was placed at room temperature for 10 min and then centrifuged in a precooled centrifuge for 15 min. The aqueous layer was aspirated, isopropanol was added, and then placed at room temperature for 10 min. After repeated centrifugation, 75% ethanol was added to suspend the precipitate, and the precipitate was vacuum dried, followed by determination of the absorbance at 260 nm (the range within 0.8–1.0 indicated good purity). Real‐time fluorescent quantitative PCR (RealMaster Mix SYBR Green kit, Tiangen) kit was further used. β‐actin was used as an internal control. The reverse transcription reaction was performed at 42°C for 30 min and inactivated at 94°C for 5 min. Afterward, cDNA was amplified for 30 cycles, each containing denaturation at 94°C for 1 min, annealing at 60°C for 1 min, and extension at 72°C for 80 s. The whole PCR reaction system was 50 µl. Finally, the relative expression of target mRNA was calculated.

### 2.9 Establishment and grouping of PD mouse model

OHDA (Sigma) was diluted into 2 µg/µl of 0.2% vitamin C–normal saline for further use. Mice were fixed on a stereotaxic apparatus after anesthesia, and 5 µl of 6‐OHDA solution was injected into each position according to previously described method in literature. Subsequently, mice were intraperitoneally injected with 10 IU of penicillin for 1 week. Mice were housed under the same environment with the same feeding. The behavioral changes of mice were observed every day after surgery: Whether there were abnormalities, including slow movement, less movement, tremor, vertical hair and olfactory abnormality. Rotation was induced by subcutaneous injection of apomorphine on the 8th day after surgery, and the number rotations within 0.5 hr after injection was recorded. The number of rotations/min over seven indicated successful model establishment. Mice were divided into sham operation group (Sham), model group (Model), and ACP group. Mice in ACP group were administered with 10, 50, and 100 mg/kg ACP once daily, while mice in Sham and Model groups were given the same amount of normal saline. Mice were kept in the same environment with the same feeding for 7 days. Animal experiments have been reviewed by the ethics committee, comply with the ethical regulations of animal experiments, and approved. The whole process of animal experiments complies with ethical codes and animal welfare regulations.

### 2.10 Detection of substantia nigra–striatum ROS levels by ROS primary fluorescence assay kit

The striatum structure of mice was isolated using the ROS primary fluorescence assay kit (Shanghai Dailong Biotechnology Co., Ltd.). Briefly, 200 mg of tissue was added to Reagent A for cleaning, cut into the broken tissue in sterile, and then added with Dilution C, followed by complete shaking. The mixture was added into the precooled Dounce homogenizer to grind the tissue, and the tissue was further transferred to the conical tube. Homogenate of 50 µl was added with 50 µl of Dilution C, followed by addition of 20 µl of the staining solution. After incubation for 20 min, a fluorescent plate reader was used for detection, and the excitation wavelength was 490 nm and the emission wavelength was 520 nm.

### 2.11 The expression level of NLRP3‐related protein in the substantia nigra

Western blot was used for protein detection, and the method was consistent with the above cellular experiments.

### 2.12 Statistical analysis

Measurement data were shown as $\bar{x}\pm s$. One‐way ANOVA was used for data comparison, and LSD analysis was used for intragroup comparison. A *p* < .05 was considered as statistical significance. SPSS 17.0 software was utilized for statistical analysis.

## 3 RESULTS

### 3.1 Results of 6‐OHDA‐activated ROS‐induced NLRP3 activation

**FIGURE 1:**
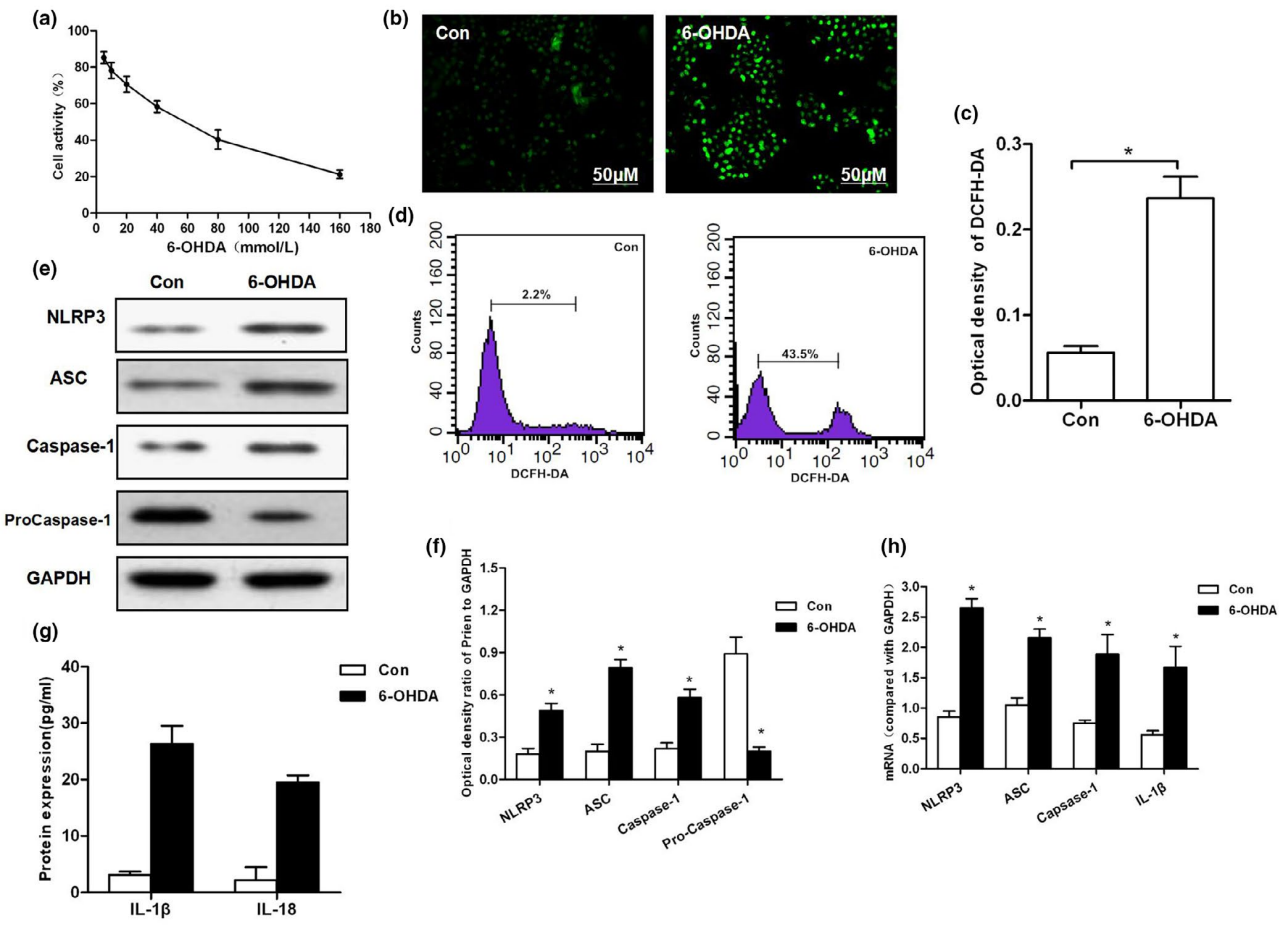
Results of 6‐OHDA‐induced ROS‐NLRP3 activation in MES23.5 cells. (a) Effect of 6‐OHDA on cell viability and IC50 values at gradient concentrations. (b, c) The levels of ROS production detected by DCFH‐DA probe and corresponding statistical results. (d) Flow cytometry was used to detect DCFH‐DA‐positive cells. 6‐OHDA could induce ROS production, and the number of positive cells was significantly higher than that of Con group (*p* < .01). (e–h) 6‐OHDA induced the activation of NLRP3 in cells. The protein and mRNA levels of key proteins, including NLRP3, ASC, and caspase‐1, were significantly increased, compared with Con group, *p < .05; levels of inflammatory factors, including IL‐1β and IL‐18, compared to Con group, *p < .05

CCK8 assay showed that cell viability was significantly reduced in response to increasing dose of 6‐OHDA, with the IC50 value of 6‐OHDA being 50 mmol/L. DCFH‐DA probe and flow cytometry revealed that 6‐OHDA could induce the production of cellular ROS. In addition, Western blot analysis showed that the increased level of ROS could further promote the expression of NLRP3, ASC, and caspase‐1; simultaneously, the protein and mRNA levels of pro‐caspase‐1 were consistently down‐regulated. Similarly, the expression levels of secretory inflammatory factors, IL‐1β and IL‐18 in the medium, were increased, indicating that 6‐OHDA could induce the activation of ROS‐NLRP3 and the damage of dopaminergic neurons (shown in Figure 1).

### 3.2 ROS inhibition decreased 6‐OHDA‐induced NLRP3 activation

**FIGURE 2:**
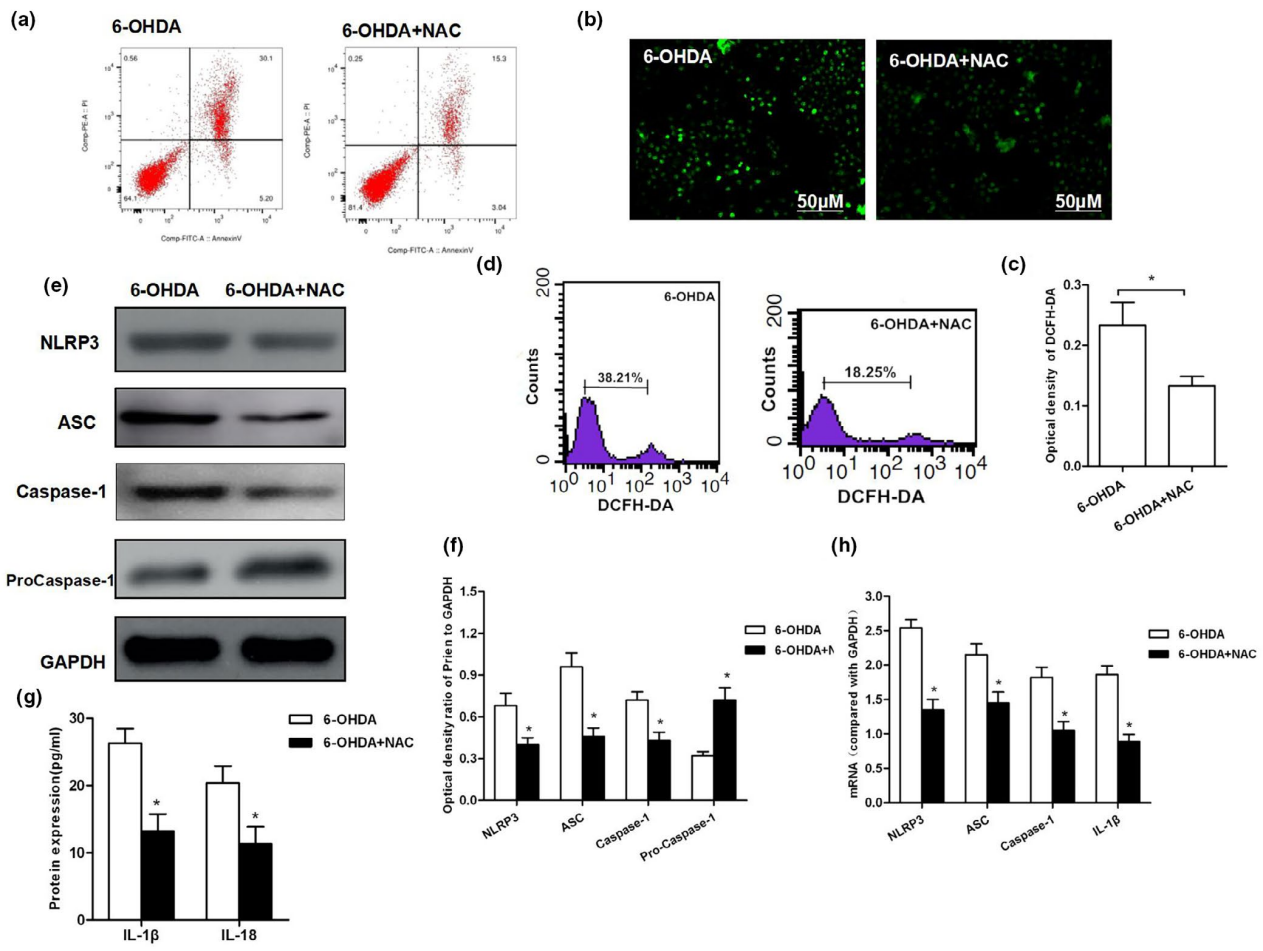
ROS inhibition resisted 6‐OHDA‐induced NLRP3 activation resulted in cell injury. (a) After inhibition of ROS, the same concentration of 6‐OHDA was used for intervention. As a result, the apoptotic rate was significantly down‐regulated in 6‐OHDA + NAC group than that in 6‐OHDA group (*p* < .05). (b, c) DCFH‐DA probe was used to detect the level of ROS production and corresponding statistical results. (d) Flow cytometry was used to detect DCFH‐DA‐positive cells. The number of positive cells was significantly less in 6‐OHDA‐NAC group than that in 6‐OHDA group (*p* < .01).(e–h) Detection of NLRP3 activation: Both protein and mRNA levels of key proteins NLRP3, ASC, and caspase‐1 were significantly decreased in 6‐OHDA‐NAC group than those in 6‐OHDA group, *p < .05; the levels of inflammatory factors IL‐1β and IL, *p < .05 compared to 6‐OHDA

Cells were pretreated with ROS inhibitor NAC, followed by intervention of 50 mmol/L of 6‐OHDA. Cell apoptotic level was significantly higher in 6‐OHDA group compared to that in 6‐OHDA + NAC group (*p* < .05). The levels of ROS, NLRP3, ASC, and caspase‐1 were significantly down‐regulated (*p* < .05), while that of pro‐caspase‐1 was higher in 6‐OHDA + NAC group in comparison with those in 6‐OHDA group (*p* < .05). In addition, both protein and mRNA levels were consistent, indicating that NLRP3 activation was inhibited. Moreover, the expression level of IL‐1β and IL‐18 in the culture medium was significantly lower in 6‐OHDA + NAC group than that of 6‐OHDA group (*p* < .05). These results suggested that ROS inhibition can significantly decrease the activation of NLRP3, thereby protecting neuronal cells (shown in Figure 2).

### 3.3 ACP inhibited ROS‐NLRP3 activation to protect dopaminergic neurons

**FIGURE 3:**
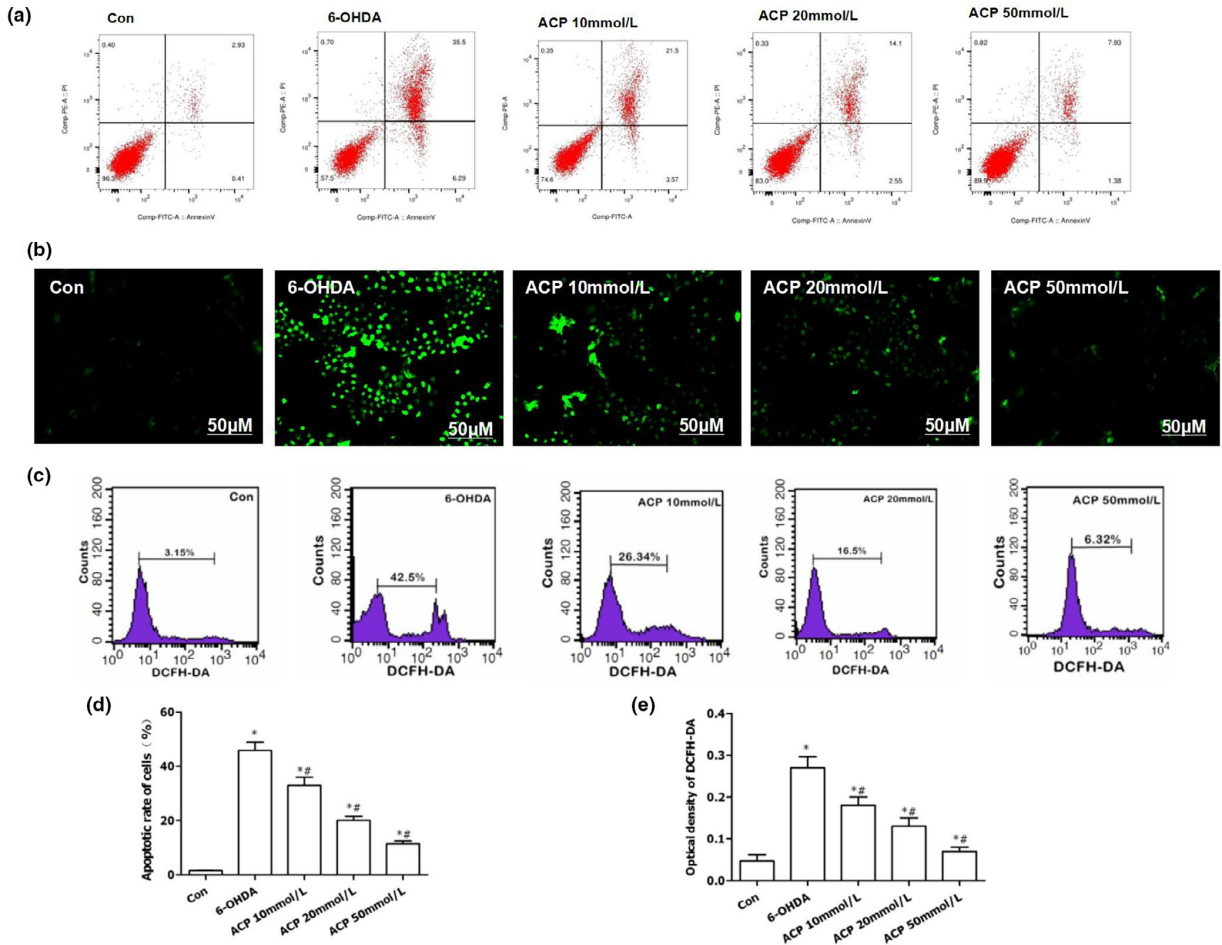
ACP protected dopaminergic neurons by inhibiting 6‐OHDA‐induced ROS expression. (a, d) ACP exerted a protective effect on 6‐OHDA‐induced dopaminergic neurons. ACP intervention led to significantly down‐regulated apoptotic rate, compared with Con group, *p < .05; compared with 6‐OHDA group, #*p* < .05. (b, c) DCFH‐DA staining was used to detect ROS expression. The cellular level of ROS was significantly down‐regulated after ACP intervention, and the number of positive cells decreased, as indicated by flow cytometry. (e) The results of fluorescence intensity after DCFH‐DA staining: The absorbance was significantly down‐regulated after ACP intervention in a dose‐dependent manner, compared with Con group, *p < .05; compared with 6‐OHDA group, #*p* < .05

**FIGURE 4:**
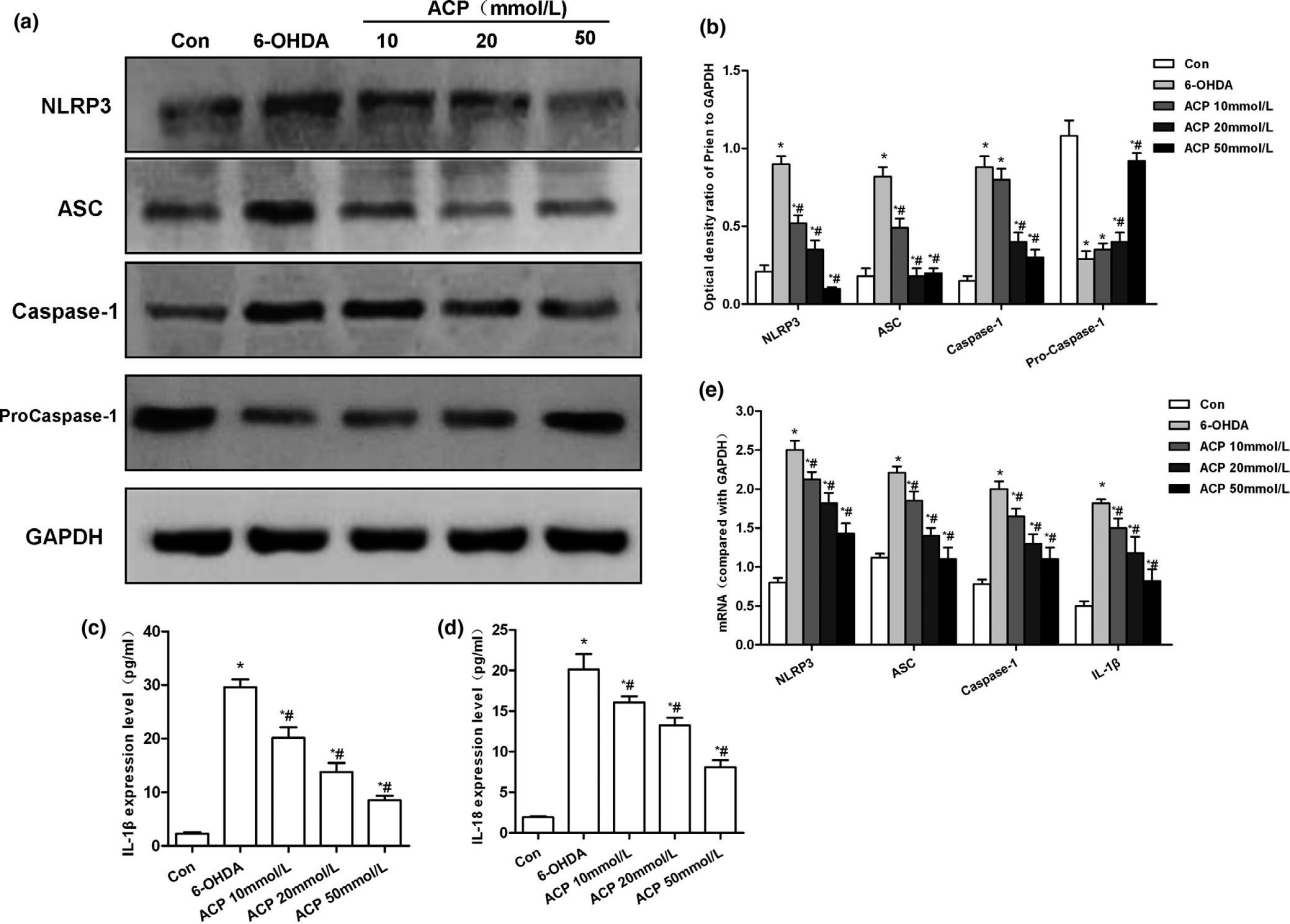
Effect of protein and mRNA expression of NPRP3 after ACP intervention. (a, b) ACP pretreatment led to significantly down‐regulated 6‐OHDA‐induced NLRP3 activation. In addition, the expression of NLRP3, ASC, and caspase‐1 was down‐regulated, while that of pro‐caspase‐1 was up‐regulated, and the level of pro‐caspase‐1 was decreased, *p < .05 compared to Con group; #*p* < .05 compared to 6‐OHDA group. (b, c) ACP pretreatment resulted in the significantly down‐regulated levels of inflammatory factors IL‐1β and IL‐18 in the culture medium, compared with Con group, *p < .05; compared with 6‐OHDA group, #*p* < .05. (d) ACP pretreatment caused down‐regulation in the mRNA expression levels of NLRP3, ASC, caspase‐1, and IL‐1β, which was consistent with protein level trend, compared with Con group, *p < .05; compared with 6‐OHDA group, #*p* < .05

The expression level of ROS was significantly low, and the apoptotic rate was significantly decreased in Con group, in comparison with those in 6‐OHDA group (*p* < .05). After ACP intervention, the level of intracellular ROS was significantly down‐regulated, the fluorescence intensity of DCFH‐DA staining was decreased, and the apoptotic rate was reduced in a dose‐dependent manner, which was significantly different from those in 6‐OHDA group (*p* < .05). Meanwhile, the number of DCFH‐DA‐positive cells was decreased (shown in Figure 3). After ACP intervention, the activation of NLRP3 inflammasome was significantly inhibited, and the expression levels of key proteins NLRP3, ASC, and caspase‐1 were significantly down‐regulated in a dose‐dependent manner compared with those in 6‐OHDA group (*p* < .05). Meanwhile, the levels of IL‐1β and IL‐18 in the cell culture medium were also significantly down‐regulated in a dose‐dependent pattern, in comparison with those in 6‐OHDA group (*p* < .05) (shown in Figure 4).

### 3.4 Effect of ACP on the expression of ROS‐NLRP3 in the striatum of PD mice

**FIGURE 5:**
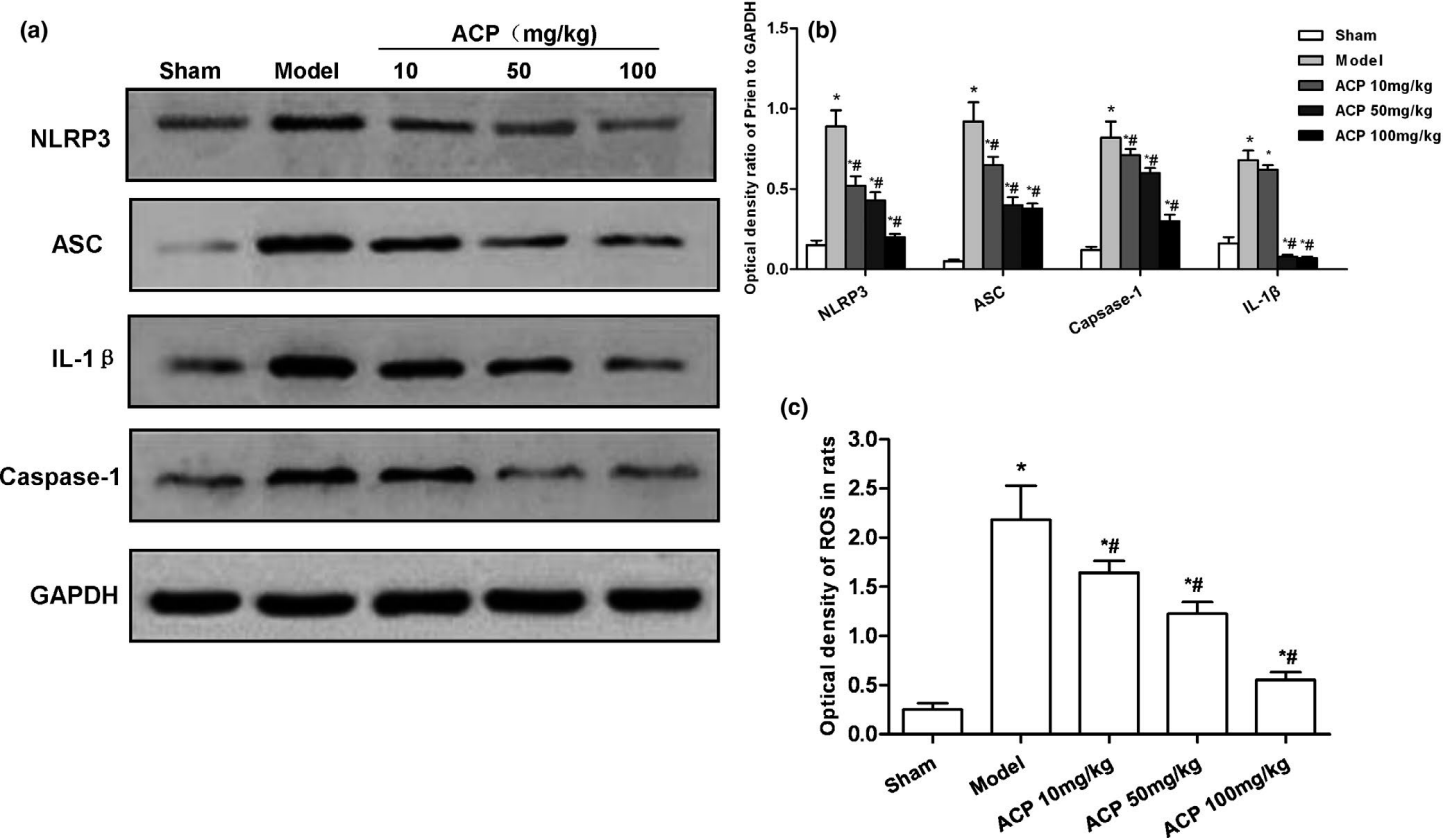
Effect of ACP on the expression of ROS‐NLRP3 in the striatum of PD mice. (a, b) ACP intervention could lead to the activation of NLRP3 inflammasome in mouse striatum. The expression levels of NLRP3, ASC, caspase‐1, and IL‐1β were significantly higher in Model group than those in Sham group. After ACP intervention, the expression level was decreased compared with Sham Group, *p < .05; compared with Model group, #*p* < .05. (c) Fluorescence detection of ROS in the striatum. The level of ROS was significantly higher in Model group than that in Sham group. Moreover, the expression level of ROS was decreased after ACP intervention, compared with Sham group, *p < .05; compared with Model group, #*p* < .05

The PD mouse model can be successfully established by 6‐OHDA injection. Mice could be induced to rotate by apomorphine, indicating the presence of PD‐like behavioral changes. ACP intervention led to significantly down‐regulated expression of ROS‐NLRP3 in the striatum. The fluorescence detection kit revealed that ROS expression was increased in the striatum after 6‐OHDA injection, which was significantly higher than that in Sham group. After ACP intervention, ROS expression was significantly reduced, which further inhibited the expression of NLRP3. Moreover, the levels of NLRP3, ASC, caspase‐1, and IL‐1β were significantly down‐regulated in the striatum in a dose‐dependent manner, indicating that ACP could significantly decrease ROS‐NLRP3 activation in the striatum (shown in Figure 5).

## 4 DISCUSSION

In recent years, a large number of studies have demonstrated that neuroinflammation plays an important role in multiple types of CNS diseases. The inflammatory response plays a locally protective response; however, long‐term inflammatory responses can lead to the pathogenesis of various neurodegenerative diseases. In PD studies, NF‐kB has been considered as a classical signaling. NF‐kB activation could promote the release of various inflammatory factors, including IL‐1β, IL‐18, and TNF‐α, enhance the inflammatory cascade (Bauernfeind et al., 2009; Mezzasoma, Antognelli, & Talesa, 2016), and cause progressive destruction and loss of dopaminergic neurons in the midbrain nigral striatum. The activation of IL‐1β requires the cleavage of caspase‐1, during which process, NLRP3 inflammasome plays an important role (Bai et al., 2018). NLRP3 is mainly composed of N‐terminal thermoprotein structure PYD, nucleotide‐binding oligomerization domain NPD, and C‐terminal domain. The NLRP3 inflammasome can serve as a compound protein molecular platform for the activation of immature caspase‐1. NLRP3 activation can further accumulate the apical protein ASC and pro‐caspase‐1, which are related to apoptosis of the adaptor protein, to form mature NLRP3 inflammasome, thereby further activating caspase‐1 to form the NLRP3/ASC/caspase‐1 inflammatory cascade pathway, to promote the maturation and secretion of pro‐inflammatory cytokines, such as IL‐1β and IL‐18 (Provoost et al., 2011; Wang et al., 2018). NLRP3 inflammasome can be activated not only by a variety of exogenous pathogens, but also by certain endogenous signals and metabolites. ROS is an upstream signal for NLRP3 activation. Studies have shown that ROS scavengers and inhibition of NADPH oxidase can extensively inhibit the activation of NLRP3 (Peng et al., 2017; Wu et al., 2018). Another study has shown that ROS can promote the binding of TXNIP to NLRP3 to activate NLRP3 (Liu et al., 2015). ROS‐mediated oxidative stress injury also exists in inflammation of CNS. Multiple studies have found that ROS activation exists in the pathogenesis and progression of neuroinflammation. Therefore, ROS‐NLRP3 signaling is involved in neuroinflammation, playing an important role in PD (Hernandes, 2014).

6‐OHDA is a common neurotoxin, with important applications in the establishment of PD models, which can damage dopaminergic neurons and cause PD‐like behavioral changes (Slominsky et al., 2017). In this study, we found that the mechanism of 6‐OHDA‐induced neuronal injury is associated with ROS‐NLRP3 signaling. 6‐OHDA can induce the production of ROS, thereby activating NLRP3 expression, and simultaneously releasing inflammatory factors, causing neuronal apoptosis. The pretreatment of NAC could inhibit ROS production, thereby resisting the activation of NLRP3 and inhibiting cell apoptosis.

Antrodia camphorata is a porous fungus unique to Taiwan, rich in polysaccharides, triterpenoids, adenosine, and other active substances. Previous studies have found that Antrodia camphorata exerts a good therapeutic effect on liver damage, tumors, and other diseases (And & Yen, 2002). Our previous studies also have found that ACP can improve the behavior of PD mice; therefore, in this study, we focused on dopaminergic neurons to further reveal the mechanism of ACP on neuron protection. As a result, the protective effect of ACP on dopamine neurons is related to the inhibition of ROS‐NLRP3 signaling. ACP could suppress ROS production, which is consistent at both cellular and animal levels. ROS inhibition can further reduce the expression of NLRP3, which is one of the mechanisms of ACP on neuron protection.

## 5 CONCLUSION

In this study, we find that 6‐OHDA‐induced dopaminergic neuron injury is associated with ROS‐NLRP3 signaling, and ACP can inhibit this signal to protect dopaminergic neurons. Thus, ACP is a promising natural drug for PD treatment, with good application prospects and research value. We think ACP may increase the activity and expression of antioxidant enzymes, thereby inhibiting the activation of ROS, but further verification is needed.

## CONFLICT OF INTEREST

No competing interests.

## AUTHOR CONTRIBUTIONS

Chenyang Han and Heping shen contributed to experimental design and operation; Yi Yang, Yongjia Sheng, and Jin Wang contributed to animal model construction and tissue acquisition; Wenyan Li and Xiaohong Zhou contributed to data processing and article writing; Li Guo contributed to article revision; Liping Zhai and Qiaobing Guan contributed to experimental design, operation guidance, and article review.

## ETHICAL APPROVAL AND CONSENT TO PARTICIPATE

The study was approved by the Ethics Committee.

## CONSENT FOR PUBLICATION

All authors approved the publication of the article.

### Peer Review

The peer review history for this article is available at https://publons.com/publon/10.1002/brb3.1824.

## Data Availability

The data that support the findings of this study are available from the corresponding author upon reasonable request.
