# Correction to “*Antrodia camphorata* polysaccharide resists 6‐OHDA‐induced dopaminergic neuronal damage by inhibiting ROS‐NLRP3 activation”

**DOI:** 10.1002/brb3.3392

**Published:** 2024-01-08

Han, C., Shen, H., Yang, Y., Sheng, Y., Wang, J., Li, W., Zhou, X., Guo, L., Zhai. L., & Guan, Q. (2020). *Antrodia camphorata* polysaccharide resists 6‐OHDA‐induced dopaminergic neuronal damage by inhibiting ROS‐NLRP3 activation. *Brain and Behavior*, 10, e01824. https://doi.org/10.1002/brb3.1824

In the published version of the article, an error occurred in Figure 5A, and the incorrect Western blot was inserted into the figure. This has been updated with the correct panel. The corrected figure appears below.

**FIGURE 5:**
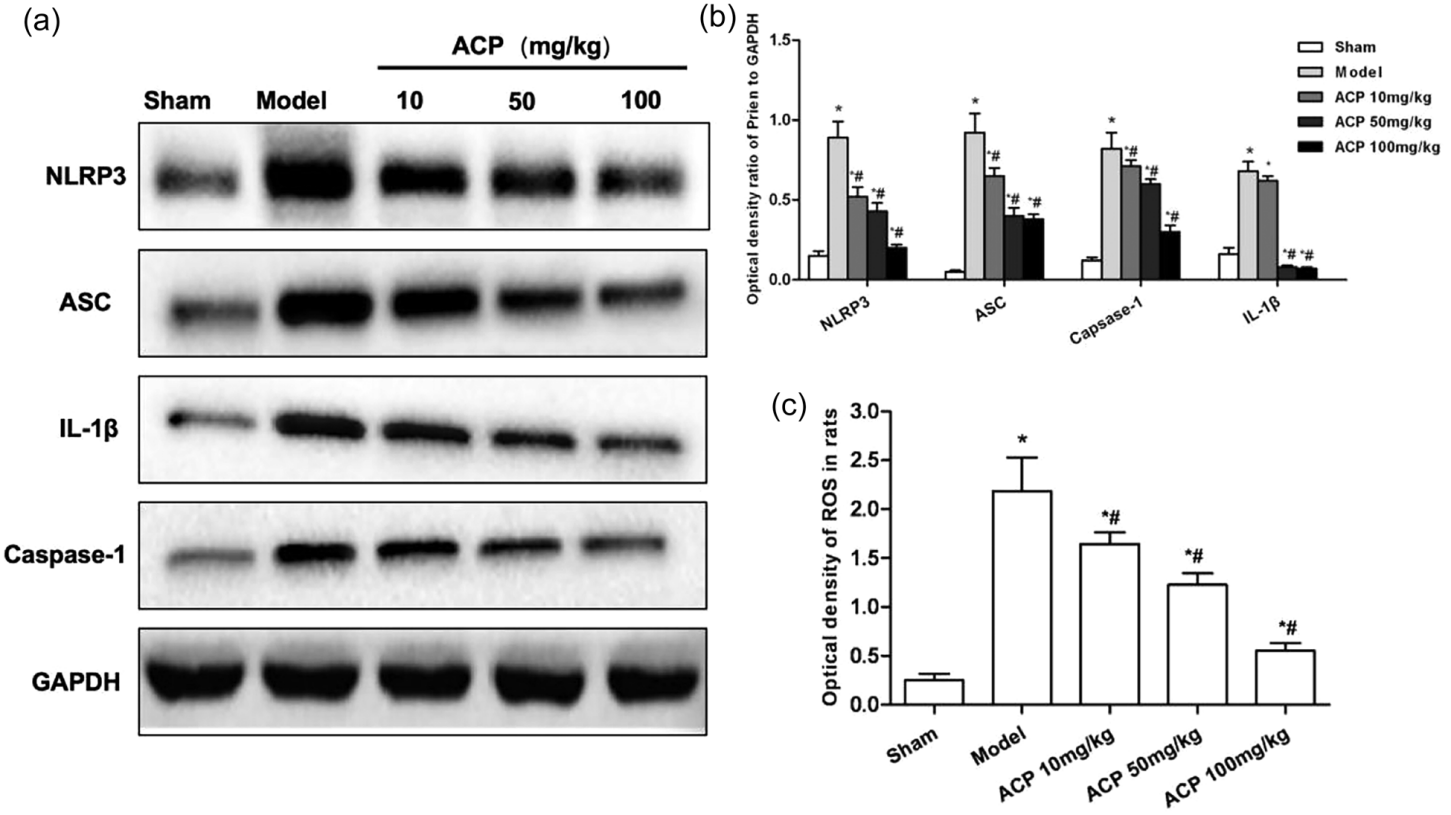


We apologize for this error.

